# Weight status change during four years and left ventricular hypertrophy in Chinese children

**DOI:** 10.3389/fped.2024.1371286

**Published:** 2024-10-25

**Authors:** Qin Liu, Cheng Li, Lili Yang, Zhuo Gong, Min Zhao, Pascal Bovet, Bo Xi

**Affiliations:** ^1^ Department of Ultrasound, Children’s Hospital of the Capital Institute of Pediatrics, Beijing, China; ^2^ Department of Epidemiology, School of Public Health, Qilu Hospital, Cheeloo College of Medicine, Shandong University, Jinan, China; ^3^ School of Public Health, Changsha Medical University, Changsha, China; ^4^ Department of Nutrition and Food Hygiene, School of Public Health, Cheeloo College of Medicine, Shandong University, Jinan, China; ^5^ Center for Primary Care and Public Health (Unisanté), University of Lausanne, Lausanne, Switzerland

**Keywords:** children, overweight, weight status change, left ventricular hypertrophy, Chinese

## Abstract

**Objective:**

It is well-established that overweight/obesity is a major risk factor for left ventricular hypertrophy (LVH) in childhood. However, it is still unclear if reversing from overweight/obesity to normal weight is associated with decreased LVH in children. This study aimed to examine the association between weight status change during four years and LVH among Chinese children based on a prospective cohort study.

**Methods:**

Data were obtained from the Huantai Childhood Cardiovascular Health Cohort Study in China. A total of 1,178 children without LVH at baseline (mean age: 8.3 years) were included in this study. According to weight status [normal weight or overweight (including obesity)] at baseline (2017) and follow-up (2021), children were divided, based on sex- and age-adjusted body mass index (BMI), into four groups: persistent normal weight (normal weight at both baseline and follow-up), incident overweight (normal weight at baseline but overweight at follow-up), reversal to normal weight (overweight at baseline but normal weight at follow-up), persistent overweight (overweight at both baseline and follow-up).

**Results:**

After adjustment for potential confounding factors, children with incident overweight (*n* = 114, 30.63 ± 4.74 g/m^2.7^) and those with persistent overweight (*n* = 363, 31.56 ± 6.24 g/m^2.7^) had higher left ventricular mass index (LVMI) at the end of the follow-up period than those with persistent normal weight (*n* = 632, 28.46 ± 7.64 g/m^2.7^), while those who reversed from overweight to normal weight had a non-significantly lower LVMI (*n* = 69, 28.51 ± 4.28 g/m^2.7^). Compared to children with persistent normal weight, those with persistent overweight [odds ratio (*OR*) = 5.14, 95% confidence interval (*CI*) = 3.33–7.95] and those with incident overweight (*OR* = 3.34, 95% *CI* = 1.77–6.30) had an increased risk of LVH. The risk of LVH tended to decrease, although not significantly, in those who reversed from overweight to normal weight (*OR* = 0.76, 95% *CI* = 0.22–2.55).

**Conclusion:**

Our findings demonstrate a positive association between overweight and left ventricular mass in children and suggest that LVH in childhood could be attenuated by weight loss.

## Introduction

Childhood overweight/obesity is a severe public health issue globally. The prevalence of overweight (including obesity) among children aged 6–17 years increased from 6.7% in 1991 to 24.4% in 2015 in China (1), and it was estimated that the total number of children with overweight would reach 50 million in China by 2030 (2). It is well-established that overweight is associated with a range of chronic diseases, such as cardiovascular diseases (CVD), several cancers, hypertension, type 2 diabetes, and several other diseases (3, 4), and overweight is also considered as a main driver of a decline in life quality and expectancy (5).

Left ventricular mass index (LVMI) and left ventricular hypertrophy (LVH) are important risk factors of cardiovascular events (6). Recent studies showed that most children with obesity are suffering from early impairment of the cardiac structure, including increased LVMI and LVH (7, 8), but evidence is mainly based on cross-sectional studies (9–11). Recently, using a longitudinal study, we described that, compared with children who had normal waist circumstance (WC) at both baseline and follow-up, those with a WC gain or with persistently elevated WC had an increased risk of LVH in childhood. However, children who reversed from abdominal obesity to normal WC did not have an increased risk of LVH (12). To our knowledge, no study has examined the effect of weight status change based on body mass index (BMI) during childhood on LVMI and the risk of LVH among Chinese children. It is still unclear whether reversal from overweight to normal weight during childhood could lower the incidence of childhood LVH. Although several studies have found that WC had better predictive effect on cardiovascular outcomes than BMI due to its ability to distinguish between fat mass and fat-free mass (13, 14), others have shown no significant difference (15). BMI is more widely used globally and has a more standardized calculation formula and classification, which may benefit more the development of child health policies. Therefore, based on a prospective cohort study of Chinese children conducted in 2017 and 2021, we examined the association between weight status change over four years and LVH among Chinese children aged 8.3 years on average at baseline.

## Methods

### Study populations

Data were from the Huantai Childhood Cardiovascular Health Cohort Study, which was conducted in one primary school from Huantai County, Zibo City, Shandong Province, China (16). The cohort is a prospective study using a convenient cluster sampling method to select eligible participants. A total of 1,516 children aged 6–11 years were recruited to participate in the baseline survey (November 2017 to January 2018), and 1,353 children of them were followed up (October to December 2021). Among them, 225 children were excluded due to loss of follow-up or missing information on anthropometric indices, ultrasound measurements, or questionnaires. We also excluded the children who had LVH at baseline (*n* = 113). Finally, 1,178 children in total were included in this study to examine the association between weight status change from 2017 to 2021 and LVMI and incident LVH. This study was approved by the Ethics Committee of School of Public Health, Shandong University (No. 20160308), and informed consent was signed by each participant and their parents/guardians.

### Physical examination

Height (cm) and weight (kg) were measured with children wearing light clothing without shoes and with 0.1 cm and 0.1 kg precision, respectively. BMI (kg/m^2^) was calculated as the ratio of weight (kg) and the square of height (m^2^).

### Cardiac ultrasound measurements

According to the Pediatric Cardiometry Guidelines of the American Society of Echocardiography (17), the structure of the left ventricle was measured by an experienced sonographer using a color Doppler ultrasound machine (S4-2, CX30; Royal Philips, Amsterdam, The Netherlands). Measurements were available on the left ventricular end-diastolic internal dimension (LVID), interventricular septal wall thickness (IVST), and left ventricular posterior wall thickness (LVPWT), and left ventricular mass (LVM) and LVMI were calculated using the following formulas: LVM (g) = 0.8 × [1.04 × (IVST + LVID + LVPWT)^3^ – (LVID)^3^] + 0.6 (18), LVMI (g/m^2.7^) = LVM (g)/height^2.7^ (m) (19).

### Definitions of weight status change and LVH

Childhood overweight (including obesity) was defined according to the sex- and age-specific BMI percentile reference for Chinese children (20). According to the weight status (normal weight vs. overweight) at baseline and follow-up, all participants were divided into four groups: persistent normal weight (normal weight at both baseline and follow-up), reversal to normal weight (overweight at baseline but normal weight at follow-up), incident overweight (normal weight at baseline but overweight at follow-up), and persistent overweight (overweight at both baseline and follow-up). In this study, LVH was defined as the LVMI ≥ the sex- and age- specific 90th percentile values based on this population (12).

### Covariates

A self-reported structured questionnaire was used to collect demographic and lifestyle information of the participants, including age, sex, parental education, dietary habits (intake of fruits/vegetables and intake of carbonated drinks), sleep duration, and physical activity. Wake-up time and bedtime were obtained by self-report, and sleep duration was calculated by subtracting bedtime from wake-up time. Physical activity (<1 vs. ≥1 h per day) and dietary intake of fruits/vegetables (<5 vs. ≥5 servings per day) were categorized according to the recommendations of World Health Organization (21, 22). We defined intake of carbonated drinks as <1 times/week vs. ≥1 times/week (23). Parental education was classified as junior high school and below vs. high school vs. college and above.

### Statistical analysis

Data on the characteristics of the participants are presented as means ± standard deviations for continuous variables and frequencies (proportions) for categorical variables. Differences in characteristics according to the four weight status groups (persistent normal weight, reversal to normal weight, incident overweight, and persistent overweight) were tested using variance analysis for continuous variables and chi-square test for categorical variables. Covariance analysis was used to assess the association between weight status change and LVMI measured at follow-up. Association between weight status change and LVH was examined using multivariate logistic regression models, and odds ratios (*OR*s) with their 95% confidence intervals (95% *CI*s) were calculated. Three models were performed to assess the confounding effects of the included covariates. Model 1 was adjusted for sex and age at baseline, and model 2 was additionally adjusted for parental education, intake of fruits/vegetables, intake of carbonated drinks, physical activity, and sleep duration at baseline. Model 3 was then additionally adjusted for intake of fruits/vegetables, intake of carbonated drinks, physical activity, and sleep duration at follow-up. All statistical analyses were conducted using SAS version 9.4 and a two-sided *P*-value <0.05 was considered to be statistically significant.

## Results

### Basic characteristics of the participants

1,178 children (males: 52.9%) were included in this study (children with LVH at baseline were not included in this study). Among them, 53.7% (632/1,178) had persistent normal weight; 5.8% (69/1,178) had overweight at baseline but normal weight at follow-up (reversal to normal weight); 9.7% (114/1,178) had normal weight at baseline but overweight at follow-up (incident overweight), and 30.8% (363/1,178) had persistent overweight at both baseline and follow-up (persistent overweight). There were significant differences in sex, age, BMI, and LVMI at baseline across the four groups. Children in the persistent overweight group tended to have a higher LVMI than those in the persistent normal weight group. Similar differences in age, BMI and LVMI across the four groups at follow-up were also observed (Table 1).

**Table 1:** Characteristics of the participants across four groups of weight status change from baseline to follow-up.

Characteristics	*N* (%)/(*M* ± *SD*)	Change of weight status				*P*
		Persistent normal weight	Reversal to normal weight	Incident overweight	Persistent overweight	
Baseline (2017)						
Age (years)	8.3 ± 1.5	8.4 ± 1.6	8.9 ± 1.3	7.8 ± 1.5*	8.2 ± 1.6	<0.001
Sleep duration (h/day)	9.3 ± 0.5	9.3 ± 0.5	9.3 ± 0.5	9.4 ± 0.5	9.3 ± 0.5	0.723
BMI (kg/m^2^)	17.7 ± 1.7	15.7 ± 1.3	19.7 ± 1.7*	16.8 ± 1.1*	20.9 ± 2.4*	<0.001
LVMI (g/m^2.7^)	27.4 ± 3.4	26.7 ± 3.3	27.1 ± 3.7	27.4 ± 3.0	28.9 ± 3.8*	<0.001
Sex						
Male	623 (52.9)	311 (49.2)	35 (50.7)	65 (57.0)	212 (58.4)	0.033
Female	555 (47.1)	321 (50.8)	34 (49.3)	49 (43.0)	151 (41.6)	
Physical activity						
<1 h/day	974 (82.7)	522 (82.6)	50 (72.5)	99 (86.8)	303 (83.5)	0.087
≥1 h/day	204 (17.3)	110 (17.4)	19 (27.5)	15 (13.2)	60 (16.5)	
Intake of fruits/vegetables						
<5 times/day	354 (30.1)	189 (29.9)	30 (43.5)	29 (25.4)	106 (29.2)	0.066
≥5 times/day	824 (69.9)	443 (70.1)	39 (56.5)	85 (74.6)	257 (70.8)	
Intake of carbonated drinks						
<1 time/week	1,109 (94.1)	597 (94.5)	63 (91.3)	109 (95.6)	340 (93.7)	0.632
≥1 times/week	69 (5.9)	35 (5.5)	6 (8.7)	5 (4.4)	23 (6.3)	
Father's education						
Junior high and below	259 (22.0)	132 (20.9)	15 (21.8)	27 (23.7)	85 (23.4)	0.677
High school	385 (32.7)	221 (35.0)	19 (27.5)	36 (31.6)	109 (30.0)	
College and above	534 (45.3)	279 (44.1)	35 (50.7)	51 (44.7)	169 (46.6)	
Mother's education						
Junior high and below	328 (27.8)	172 (27.2)	21 (30.4)	34 (29.8)	101 (27.8)	0.426
High school	353 (30.0)	203 (32.1)	17 (24.7)	25 (21.9)	108 (29.8)	
College and above	497 (42.2)	257 (40.7)	31 (44.9)	55 (48.3)	154 (42.4)	
Follow-up (2021)						
Age (years)	12.2 ± 1.5	12.3 ± 1.5	12.8 ± 1.3	11.7 ± 1.4*	12.1 ± 1.5	<0.001
Sleep duration (h/day)	8.5 ± 1.0	8.5 ± 1.0	8.1 ± 1.0*	8.7 ± 0.9	8.5 ± 1.0	0.001
BMI (kg/m^2^)	20.8 ± 2.2	18.0 ± 1.9	20.2 ± 1.2*	22.2 ± 1.6*	25.3 ± 2.9*	<0.001
LVMI (g/m^2.7^)	29.5 ± 3.8	28.3 ± 3.5	28.6 ± 3.3	30.4 ± 3.2*	31.5 ± 4.5*	<0.001
Physical activity						
<1 h/day	362 (30.7)	212 (33.5)	17 (24.6)	38 (33.3)	95 (26.2)	0.059
≥1 h/day	816 (69.3)	420 (66.5)	52 (75.4)	76 (66.7)	268 (73.8)	
Intake of fruits/vegetables						
<5 times/day	268 (22.8)	144 (22.8)	24 (34.8)	19 (16.7)	81 (22.3)	0.044
≥5 times/day	910 (77.2)	488 (77.2)	45 (65.2)	95 (83.3)	282 (77.7)	
Intake of carbonated drinks						
<1 time/week	991 (84.1)	543 (85.9)	59 (85.5)	94 (82.5)	295 (81.3)	0.253
≥1 times/week	187 (15.9)	89 (14.1)	10 (14.5)	20 (17.5)	68 (18.7)	

* *P* < 0.05 vs. persistent normal weight.

### Weight status change and the levels of childhood LVMI

After adjustment for all potential confounding factors, LVMI (at follow-up) of children in the incident overweight group (*n* = 114, 30.63 ± 4.74 g/m^2.7^) and persistent overweight group (*n* = 363, 31.56 ± 6.24 g/m^2.7^) were significantly higher than those in persistent normal weight group (*n* = 632, 28.46 ± 7.64 g/m^2.7^), whereas LVMI of children who reversed from overweight to normal weight (*n* = 69, 28.51 ± 4.28 g/m^2.7^) was not statistically different from the persistent normal weight group, recognizing that the sample size in this group is very small (Table 2).

**Table 2:** Association between the change in weight status and LVMI (g/m^2.7^) among Chinese children.

Weight status change	Model 1		Model 2		Model 3	
	*M* ± *SD*	*P* ^b^	*M* ± *SD*	*P* ^b^	*M* ± *SD*	*P* ^b^
Persistent normal weight (*n* = 632)	28.33 ± 3.74	Reference	28.41 ± 7.02	Reference	28.46 ± 7.64	Reference
Reversal to normal weight (*n* = 69)	28.41 ± 3.75	0.860	28.44 ± 4.14	0.933	28.51 ± 4.28	0.912
Incident overweight (*n* = 114)	30.47 ± 3.75	<0.001	30.57 ± 4.59	<0.001	30.63 ± 4.74	<0.001
Persistent overweight (*n* = 363)	31.43 ± 3.75	<0.001	31.49 ± 5.83	<0.001	31.56 ± 6.24	<0.001
*P* ^a^	<0.001		<0.001		<0.001	

Model 1: Adjusted for sex, age at baseline.

Model 2: Model 1 + additionally adjusted for sleep duration, physical activity, intake of fruit/vegetables, intake of carbonated drinks and parental education levels at baseline.

Model 3: Model 2 + additionally adjusted for sleep duration, physical activity, intake of fruit/vegetables, and intake of carbonated drinks at follow-up.

^a^ *P*-value for difference in LVMI levels across four groups of weight status change.

^b^ *P*-value for difference in LVMI levels between the persistent normal weight group and the specific group.

### Weight status change and childhood LVH

**Figure 1:**
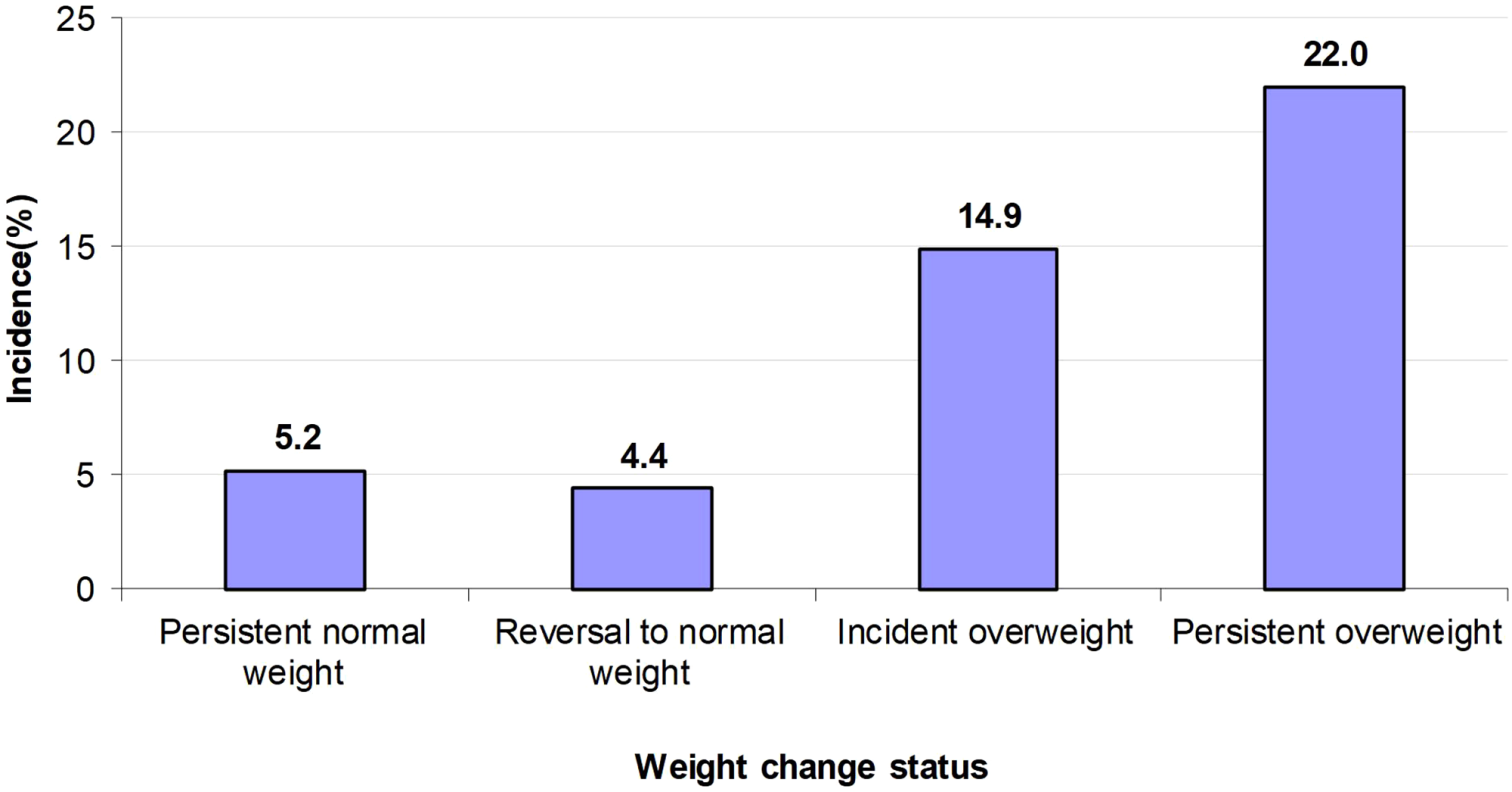
Incident rates of LVH across four groups of weight change status.

The incidence rates of LVH over the 4-year follow-up period were higher in the persistent overweight group (22.0%) and the incident overweight group (14.9%) than those in the persistent normal weight group (5.2%) and reversal to normal weight group (4.4%) (Figure 1). Compared with children who had persistent normal weight, those who had persistent overweight (*OR* = 5.14, 95% *CI* = 3.33–7.95) and incident overweight (*OR* = 3.34, 95% *CI* = 1.77–6.30) had higher odds of LVH. In contrast, the few children (*n* = 69) who reversed from overweight to normal weight had lower but statistically non-significant odds of LVH (*OR* = 0.76, 95% *CI* = 0.22–2.55) (Table 3).

**Table 3:** Association between the change in weight status and LVH among Chinese children.

Weight status change	Incident rate of LVH (%)	Model 1		Model 2		Model 3	
		*OR* (95% *CI*)	*P*	*OR* (95% *CI*)	*P*	*OR* (95% *CI*)	*P*
Persistent normal weight (*n* = 632)	5.22	Reference		Reference		Reference	
Reversal to normal weight (*n* = 69)	4.35	0.82 (0.25, 2.76)	0.751	0.78 (0.23, 2.62)	0.686	0.76 (0.22, 2.55)	0.651
Incident overweight (*n* = 114)	14.91	3.22 (1.72, 6.04)	<0.001	3.31 (1.76, 6.23)	<0.001	3.34 (1.77, 6.30)	<0.001
Persistent overweight (*n* = 363)	22.04	5.18 (3.37, 7.99)	<0.001	5.21 (3.38, 8.04)	<0.001	5.14 (3.33, 7.95)	<0.001

CI, confidence interval; OR, odds ratio.

Model 1: Adjusted for sex, age at baseline.

Model 2: Model 1 + additionally adjusted for sleep duration, physical activity, intake of fruit/vegetables, intake of carbonated drinks and parental education levels at baseline.

Model 3: Model 2 + additionally adjusted for sleep duration, physical activity, intake of fruit/vegetables, and intake of carbonated drinks at follow-up.

## Discussion

To our knowledge, this is the first study that assessed the association between weight status change during childhood and childhood LVH. Our study showed that children who developed overweight during a four-year period or maintained overweight had an increased risk of LVH compared with those who had persistent normal weight. In addition, children who reversed from overweight to normal weight status had a slightly smaller, although not significant, risk of LVH.

Previous studies had assessed the association between weight change from childhood to adulthood and left ventricular structure in adulthood (24, 25). For instance, the data from the Beijing Blood Pressure Cohort Study showed that the cumulative burden of overweight from childhood to adulthood was independently associated with the risk of LVH and abnormal left ventricular geometric patterns (including concentric remodeling, eccentric hypertrophy, and concentric hypertrophy) (25). Another multiethnic longitudinal cohort study with a mean follow-up of 28 years showed that increasing BMI from childhood to adulthood was associated with the LVMI and risk of LVH in middle-aged adulthood (24). More importantly, some studies showed that reversing weight status from overweight to normal from childhood to adulthood can reduce the risk of LVH in adulthood (5, 12), which is consistent with our findings. One longitudinal study with a 22.9-year follow-up in China showed that compared with participants who had persistent normal weight from childhood to adulthood, those who had persistent overweight (*OR* = 9.58, 95% *CI* = 5.92–15.49) and those who had incident overweight (*OR* = 5.96, 95% *CI* = 4.01–8.86) had a higher risk of LVH in adulthood. However, the few individuals (*n* = 22) who were overweight in childhood but had a normal weight in adulthood did not seem to have a markedly increased risk of LVH (*OR* = 2.43, 95% *CI* = 0.85–6.95) (26). Those findings above underscore the importance of maintaining a normal weight throughout the life course and suggest that individuals who were overweight in childhood but reversed to a normal weight later can decrease their risk of LVH, although likely not to levels as low as those who maintained a normal weight at all times.

LVH is usually regarded as a robust predictor of CVD events (27). Previous studies suggested that the relationship between weight status change and CVD events showed similar features as discussed above, which further support our findings. For example, a pooled analysis of four cohort studies reported that compared with individuals who had a normal weight from childhood to adulthood, those who were overweight in adulthood irrespective of their weight status in childhood had a higher risk of CVD in adulthood, whereas those who were overweight in childhood but non-overweight in adulthood did not have a higher risk of CVD (4). As for other risk factors of CVD in adulthood used for outcomes, a meta-analysis showed that those who had a normal weight in childhood but were overweight in adulthood or those with persistent overweight from childhood to adulthood had an increased risk of high carotid intima-median thickness, type 2 diabetes, dyslipidemia, and hypertension in adulthood (5). However, those who were overweight in childhood but not in adulthood had largely decreased risk. Those data suggest a large potential to reduce CVD risk when controlling reversing from overweight to normal weight.

All these studies above, including ours, indicated the adverse influence of overweight/obesity on childhood cardiovascular health and highlighted the importance of losing weight for individuals who were currently overweight/obese. Yet, only very few children (*n* = 69, 5.8%) managed to lose weight in our study, which is consistent with the well-known challenge for individuals to lose weight without intensive behavioral therapy or treatment (28). In contrast, a relatively great number (*n* = 114, 9.7%) of individuals changed from normal weight to overweight/obesity, which poses a great challenge to the prevention of cardiovascular outcomes. Therefore, the government and families are encouraged to strengthen the management of children's body weight to decrease the potential cardiovascular burden.

Usually, children with overweight/obesity tend to choose the healthy way of changing their lifestyle habits when they are asked to lose weight. Previous studies found that adopting healthy lifestyles (e.g., eating healthy diets and doing more exercise) among individuals with overweight/obesity can significantly improve their heart structure and function (29–31). This may explain the reasons why children who reverse from overweight/obesity to normal weight have a lower risk of LVH. A meta-analysis that included 1,022 obese adults who underwent bariatric surgery showed that this therapeutic approach can contribute to the regression of LVH and improvement of diastolic function (32). Another study of 38 morbidly obese adolescents aged 13–19 years showed that weight loss was significantly correlated with a decrease in LVMI after bariatric surgery (33). Despite there are a variety of approaches to losing weight, ultimately all the results suggest that weight loss has a beneficial effect on cardiovascular health in childhood. Those results remind children who are overweight or obese to lose weight and try to reverse to a normal weight as soon as possible for the prevention of cardiovascular damage.

Although this study is among the first to convincingly report the association between weight status change and LVH among children, several limitations of this study should be mentioned. First, all children were selected from one primary school, so the generalizability of our findings should be cautious. Second, all the participants were followed up for only four years, further studies with longer duration are needed to confirm our findings. Third, the sample size of this study is not large, particularly in the group of children who were overweight/obesity at baseline and lost weight later, and the low statistical power impeded us from performing further subgroup analysis by participants’ characteristics (e.g., sex, age, etc.). Fourth, we combined the overweight and obesity due to the relatively small sample sizes in the subgroups, which may interfere with the consolidated conclusion. Therefore, a longitudinal cohort design with a larger sample size to assess the specific effect of overweight and obesity is needed to further validate our conclusions.

## Conclusions

In summary, we found that children who had persistent overweight or incident overweight over a four-year period had an increased risk of LVH in childhood. Conversely, the risk of acquiring LVH was lower among the few children who reversed from overweight to normal weight. These findings highlight the importance of maintaining a normal weight over the life course but also suggest a reduced risk of LVH for those who are overweight or obese but succeed in normalizing their weight.

## Data Availability

The raw data supporting the conclusions of this article will be made available by the authors, without undue reservation.
